# Prevalence of Rheumatic Heart Disease and Other Cardiac Conditions in Low-Risk Pregnancies in Kenya: A Prospective Echocardiography Screening Study

**DOI:** 10.5334/gh.826

**Published:** 2021-02-09

**Authors:** John W. Snelgrove, Joy Marsha Alera, Michael C. Foster, Kipchumba C. N. Bett, Gerald S. Bloomfield, Candice K. Silversides, Felix A. Barasa, Astrid Christoffersen-Deb, Heather C. Millar, Julie G. Thorne, Rachel F. Spitzer, Rajesh Vedanthan, Nanette Okun

**Affiliations:** 1 Division of Maternal-Fetal Medicine, Mount Sinai Hospital, University of Toronto, Toronto, Ontario, CA; 2 Department of Obstetrics and Gynaecology, Mount Sinai Hospital, University of Toronto, Toronto, Ontario, CA; 3 Directorate of Reproductive Health, Moi Teaching and Referral Hospital, Eldoret, KE; 4 Academic Model Providing Access to Healthcare (AMPATH), Eldoret, KE; 5 Heart Center, Duke University Medical Center, Durham, NC, US; 6 Department of Medicine, Duke Clinical Research Institute and Duke Global Health Institute, Duke University, Durham, NC, US; 7 Division of Cardiology, Department of Medicine, Mount Sinai Hospital/Toronto General Hospital, University of Toronto, Toronto, ON, CA; 8 Department of Cardiology, Moi Teaching and Referral Hospital, Eldoret, KE; 9 Department of Obstetrics and Gynaecology, BC Children’s Hospital, University of British Columbia, Vancouver, BC, CA; 10 Section for Global Health, Department of Population Health, New York University School of Medicine, New York, NY, US

**Keywords:** rheumatic heart disease, pregnancy, echocardiography, epidemiology, Kenya, Africa

## Abstract

**Background:**

Rheumatic heart disease (RHD) in sub-Saharan Africa contributes to significant cardiac morbidity and mortality, yet prevalence estimates of RHD lesions in pregnancy are lacking.

**Objectives:**

Our first aim was to evaluate women using echocardiography to estimate the prevalence of RHD and other cardiac lesions in low-risk pregnancies. Our second aim was to assess the feasibility of screening echocardiography and its acceptability to patients.

**Methods:**

We prospectively recruited 601 pregnant women from a low-risk antenatal clinic at a tertiary care maternity centre in Western Kenya. Women completed a questionnaire about past medical history and cardiac symptoms. They underwent standardized screening echocardiography to evaluate RHD and non-RHD associated cardiac lesions. Our primary outcome was RHD-associated cardiac lesions and our secondary outcome was a composite of any clinically-relevant cardiac lesion or echocardiography finding. We also recorded duration of screening echocardiography and its acceptability among pregnant women in this sample.

**Results:**

The point prevalence of RHD-associated cardiac lesions was 5.0/1,000 (95% confidence interval: 1.0–14.5), and the point prevalence of all clinically significant lesions/findings was 21.6/1,000 (11.6–36.7). Mean screening time was seven minutes (SD 1.7, range: 4–17) for women without cardiac abnormalities and 13 minutes (SD 4.6, range: 6–23) for women with abnormal findings. Echocardiography was acceptable to women with 74.2% agreeing to participate.

**Conclusions:**

The prevalence of clinically-relevant cardiac lesions was moderately high in a low-risk population of pregnant women in Western Kenya.

## 1. Introduction

Rheumatic heart disease (RHD) is a chronic cardiac condition associated with valvular dysfunction that can significantly complicate pregnancy. Pregnant women with RHD are more likely to experience arrhythmias, congestive heart failure, hospitalization, and death [1,2]. Fetal and neonatal outcomes are significantly worse in pregnancies complicated by RHD with elevated risks of preterm birth, intrauterine growth restriction, low birthweight, and perinatal death [1].

RHD is no longer a significant cause of heart disease in high-income countries resulting from advances made in treating childhood streptococcal pharyngitis [3]. Rheumatic fever and its clinical sequelae now disproportionately affect those living in low-income countries. Sub-Saharan Africa in particular had an estimated RHD prevalence between 4–10 cases per 1,000 in the decade leading up to 2000 and the RHD-associated mortality rate increased between 1990 and 2013 [3,4]. Research with enhanced case finding using echocardiography suggests that the true prevalence may be ten-fold higher in some countries [5]. In addition, adverse outcome rates in pregnancies affected by RHD are elevated in low-income settings. For example, a retrospective study from Senegal reported a maternal mortality rate of 34% associated with RHD along with a neonatal mortality rate above 7% [6].

While this once ubiquitous cardiac disease now predominantly affects low-income countries, studies on RHD prevalence in pregnancy are lacking in sub-Saharan Africa [7]. Knowing the prevalence rate of RHD in pregnancy would help inform health policy related to population screening and management in efforts to reduce adverse maternal and perinatal outcomes.

The aims of this study were:

1. To determine the prevalence of RHD-associated and other cardiac lesions in low-risk pregnancies by offering on-site echocardiography screening prospectively to women seeking antenatal care at a tertiary maternity hospital in Western Kenya.
2. To evaluate the time required for screening echocardiography and the acceptability among patients of integrating echocardiography as a screening tool for RHD and cardiac disease in pregnancy.

## 2. Methods

### 2.1 Setting and study sample

Moi Teaching and Referral Hospital (MTRH) is a tertiary maternity hospital and regional referral centre in Eldoret, Kenya with 1,200 deliveries per month. Severe maternal morbidity due to cardiac disease occurs with a monthly incidence of 3–4 cases. A large retrospective review from MTRH showed that one-third of maternal deaths were due to indirect causes including cardiac disease [8]. Acute exacerbations of cardiac disease are among the most clinically challenging conditions for the obstetrical team to manage, and account for a maternal mortality rate of 8% at MTRH [9]. Currently, there is no routine cardiac screening in the MTRH antenatal clinics. The clinics are divided to provide low- and high-risk antenatal care.

Pregnant women at any gestational age presenting to MTRH for routine antenatal care were invited to participate in this study. We limited recruitment to the low-risk clinics to avoid including women with diagnosed cardiac disease as these women may have been referred for tertiary care at MTRH and represent a selected sample. As this study was aimed at estimating the population point prevalence, women were eligible if they had symptoms or past medical history suspicious for cardiac disease without a formal cardiac diagnosis. Women who were medically unstable to undergo echocardiography or who were not able to give consent were not eligible for the study. Women were invited to enroll and informed consent was obtained by a Kiswahili-speaking local research assistant in the waiting area for the low-risk antenatal clinics. Paper copies of consent were retained and subjects were informed that they could withdraw from the study at any time. Recruitment extended from January to March 2018.

### 2.2 Baseline characteristics and health measures assessment

Women who agreed to participate were given a short questionnaire on baseline demographics, past medical conditions, and cardiac symptoms. We obtained information on the pregnancy, current and past medical conditions, cardiac history if known, and medication use. The research nurse recorded associated health measures: weight, height, and vital signs (temperature, heart rate, blood pressure, and oxygen saturation). De-identified answers to questionnaire items and health measures were entered into a password-protected REDCap database (version 8.1.9) [10].

### 2.3 Echocardiography assessment

Echocardiography screening criteria were developed prior to study commencement. Four cardiologists (FAB, GSB, CKS, RV) and one cardiac sonographer (MCF) developed a sonographic RHD case definition using the World Heart Federation echocardiographic criteria for definite RHD cases [11]. Additional non-RHD cardiac lesions and findings considered to be clinically relevant were also identified (Appendix 1). An assessment worksheet was then developed and trialled on nurse volunteers prior to commencement of study recruitment (Appendix 2). The focused echocardiography assessment comprised of 2D colour Doppler and spectral Doppler using a GE Healthcare Vivid Q laptop machine with a 2–4Mhz transducer. Protocol images plus any additional images deemed relevant were recorded. The echocardiography assessments were performed by an accredited Registered Cardiac Sonographer (MCF) who has both academic referral centre experience (Duke University) and previous experience working with the Cardiology department at MTRH. Echocardiography screening was performed in a designated, private space in the hospital antenatal clinic area. Women with abnormal echocardiography findings were referred to the cardiac clinic and the high-risk antenatal clinic for ongoing diagnostic workup and management. De-identified echocardiography parameters were entered into the REDCap database (version 8.1.9) [10].

### 2.4 Outcomes and sample size calculation

Our primary outcome measure was the point prevalence rate of RHD-associated cardiac lesions. Previous echocardiography screening studies in school children found prevalence rates between 20–30/1,000 in Cambodia, Mozambique [5], and Kenya [12]. One study in Eritrean pregnant women found a rate of 23.0/1,000 [13]. Using the estimated prevalence *P* = 0.025 with precision set at *P*/2 = 0.0125 and an alpha error level of 0.05, a sample size of 600 was required to observe a prevalence estimate of similar scale in our study population. The secondary outcome was any clinically-relevant cardiac lesion or echocardiography finding. The secondary outcome included RHD-associated lesions and non-RHD lesions. We measured elapsed time to perform echocardiography and recorded enrollment rates for eligible participants.

### 2.5 Analysis

Summary statistics for baseline demographic, pregnancy, and previous medical history variables were calculated for the study population. We calculated RHD-associated and other cardiac lesion prevalence rates per 1,000 and estimated exact 95% confidence intervals using the Clopper-Pearson method for binomial distributions [14]. We explored bivariable associations between baseline health measures, presence of symptoms, and medical history and the outcome of abnormal echocardiography using Student’s T tests for continuous covariates and chi-square tests for categorical covariates. To correct for multiple comparisons, we used the Benjamini-Hochberg method to control for the false discovery rate [15]. Analyses were performed using STATA 13 (StataCorp. College Station, TX).

### 2.6 Consent and ethical approval

Informed consent was obtained from each patient and the study protocol conforms to the ethical guidelines of the 1975 Declaration of Helsinki as reflected in *a priori* approval by the Research Ethics Board at Mount Sinai Hospital (Toronto, Canada, reference number 16-0205-E) and by ethics review committees at Moi University (Eldoret, Kenya), and Duke University (Durham, USA). This study was funded by departmental grants from Mount Sinai Hospital, Toronto, and the University of Toronto. The funding sources did not have any role in the design, data collection, analysis, interpretation, or reporting of the study. The corresponding author (JWS) had full access to all the data in the study and takes final responsibility for the decision to submit for publication. Data sharing agreements prohibit the publication of the study dataset; however, access may be granted to those who meet pre-specified criteria for confidential access by contacting the corresponding author.

## 3. Results

**Figure 1:**
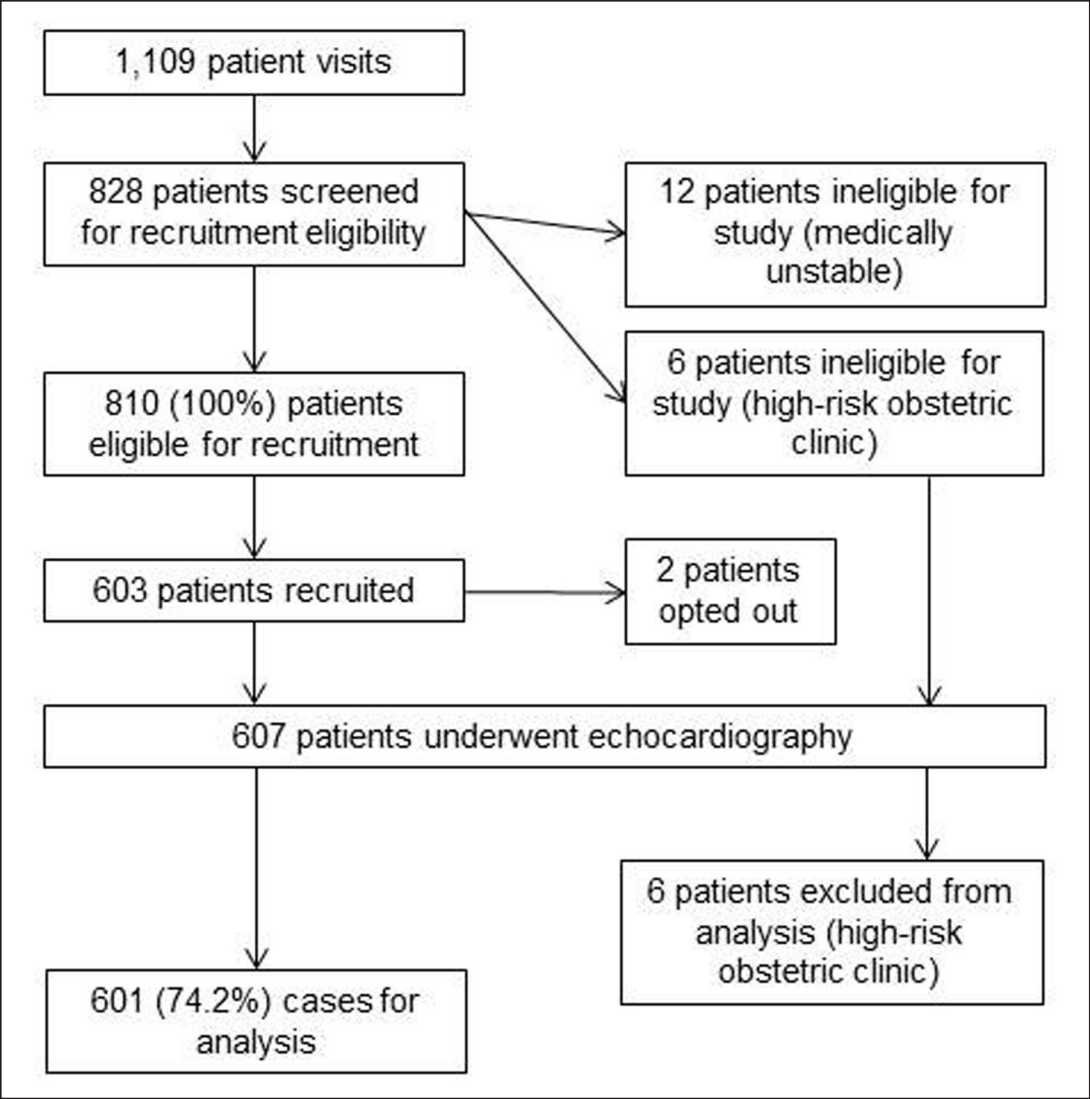
Study inclusion/exclusion flow diagram.

A total of 828 patients were screened for eligibility, 12 of whom were medically unstable and were ineligible for recruitment (Figure 1). Of the 810 women who were eligible for recruitment, 603 consented and were enrolled in the study. Two (2) women who were enrolled withdrew after recruitment, leaving a total of 601 women (74.2%) who underwent echocardiography and who represent the sample for the analyses. Six women (6) were inadvertently recruited from the high-risk antenatal clinics and, thus, did not meet our pre-specified inclusion criteria. These women underwent screening echocardiography; however, their results were excluded prior to analysis. Table 1 presents summary statistics for the demographic, pregnancy, and previous medical history variables for the 601 women included in the study. The average age was 26.6 years (standard deviation [SD] 5.7) and the average gestational age (GA) at assessment was 25.3 weeks (SD 9.5). Self-reported history suspicious for a cardiac condition was identified by 4.5% of women. None of the women in the study were able to characterize the type of condition and none had a formal cardiac diagnosis. History of rheumatic fever was endorsed by 2.0% of the total sample.

**Table 1:** Demographic and medical characteristics of study population (N = 601 women).

Characteristic	No.	Percent	Mean	SD

*Demographics*				
Age			26.6	5.7
Gravida			2.2	1.4
Parity			1.0	1.3
Highest education level				
None	20	3.3		
Primary	111	18.5		
Secondary	248	41.3		
Diploma	140	23.3		
University degree	82	13.6		
Occupation				
Unemployed	38	6.3		
Homemaker	168	28.0		
Student	85	14.1		
Employed	310	51.6		
Married/cohabitating	568	94.5		
Health insurance	297	49.4		
*Current pregnancy*				
Gestational age, GA (weeks)			25.3	9.5
GA at first visit (weeks)			17.4	7.2
Medications				
CCB	3	0.5		
HAART	45	7.5		
Other	21	3.5		
*Previous medical history*				
Suspected cardiac condition	27	4.5		
Rheumatic fever history	12	2.0		
Any non-cardiac condition	97	16.2		
Diabetes	1	0.2		
Anaemia	19	3.2		
Chronic hypertension	1	0.2		
Preeclampsia	6	1.0		
Pulmonary condition	15	2.5		
Thyroid condition	1	0.2		
Sickle cell disease	1	0.2		
HIV	45	7.5		
Other condition	18	3.0		
Mid-trimester loss	32	5.3		

Table 2 presents the case frequencies and prevalence estimates of echocardiography-identified conditions and lesions. Three women had echocardiographic evidence of RHD-associated lesions for an overall point prevalence of 5.0 per 1,000 pregnant women (95% confidence interval [CI] 1.0–14.5). One woman had aortic valve morphologic features of RHD with moderate aortic regurgitation (AR). Two women had mitral valve morphologic features of RHD and severe mitral stenosis (MS) as well as evidence of pulmonary arterial hypertension (PAH). One of these women had moderate mitral regurgitation (MR) and tricuspid regurgitation (TR) and the other had severe TR. Ten women had other clinically-relevant non-RHD cardiac lesions for a point prevalence of 16.7 per 1,000 (95% CI 8.0–30.4). Of these, valvular lesions were predominant: five cases of moderate TR and one case of moderate MR. There were two cases of bicuspid aortic valve (BAV) with mild AR, one case of patent ductus arteriosus (PDA), and one case of unroofed coronary sinus. No cases of other septal defects, cardiomyopathy, or chamber mass were identified. The total prevalence of any clinically-relevant cardiac lesion was 21.6 per 1,000 (95% CI 11.6–36.7).

**Table 2:** Case frequency and prevalence of RHD and other cardiac lesions (N = 601 women).

Echocardiography findings	No. cases (%)	Prevalence^b^ (95% CI)

RHD-associated lesions^a^	3 (0.5)	5.0 (1.0–14.5)
severe MS, severe TR, PAH	1	
severe MS, moderate MR+TR, PAH	1	
moderate AR	1	
Non-RHD lesions	10 (1.7)	16.7 (8.0–30.4)
moderate TR	5	
moderate MR	1	
BAV, mild AR	2	
PDA	1	
unroofed coronary sinus	1	
Total clinically-relevant lesions	13 (2.2)	21.6 (11.6–36.7)

^a^ Lesions that meet RHD case definition. ^b^ Prevalence per 1,000 pregnant women.

Women with any cardiac lesion(s) were significantly more likely to have a suspected, but undiagnosed history of cardiac disease (Table 3). Of the three women with RHD, two were aware they may have an undiagnosed cardiac condition based on questionnaire results. The presence of cardiac symptoms was not associated with higher likelihood of cardiac lesions. Age, body mass index (BMI), history of other medical conditions, mid-trimester loss between 20–28 weeks’ gestation and vital sign parameters were also not associated with the presence of cardiac lesions.

**Table 3:** Medical history and vital signs of women with and without cardiac lesions (N = 601).

Characteristic	Cases (n = 13)	Non-cases (n = 588)	p-value

Age (mean, SD)	25.4 (4.87)	26.7 (5.69)	0.43
BMI, m^2^/kg (mean, SD)	24.3 (3.21)	25.8 (4.52)	0.24
Symptomatic^a^ (n, %)	2 (15.4)	60 (10.2)	0.54
*Previous medical history*			
Rheumatic fever (n, %)	1 (7.7)	11 (1.9)	0.14
Suspected cardiac condition (n, %)	3 (23.1)	24 (4.1)	0.001*
Other medical condition (n, %)	2 (15.3)	95 (16.2)	0.94
Mid-trimester loss (n, %)	0 (0)	32 (5.4)	0.49
*Vital signs*			
HR (mean, SD)	85 (9.8)	89 (13.0)	0.31
sBP (mean, SD)	98 (12.0)	106 (13.4)	0.03
dBP (mean, SD)	73 (18.2)	70 (9.9)	0.25
SaO_2_, % (mean, SD)	94.3 (7.5)	95.7 (2.0)	0.05

^a^ Any chest pain, shortness-of-breath, palpitations, vertigo, light-headedness. * Statistically significant following Benjamini-Hochberg correction for multiple comparisons.

Of all eligible women invited to participate in the study, 74.2% consented, enrolled, and underwent echocardiography (Figure 1). The average time to perform a screening echo for women without cardiac disease was seven minutes (SD 1.7, range: 4–17). For women with lesions or significant findings the average echo time was 13 minutes (SD 4.6, range: 6–23).

## 4. Discussion

### 4.1 Main findings and interpretation

Rheumatic heart disease disproportionately affects young adults in settings with poor access to care; it accounts for the majority of heart disease in Kenyans under the age of 50 and is second only to congenital heart disease as the leading indication for cardiology referrals of pregnant women [16,17,18]. In Kenya, RHD is responsible for over 70% of newly diagnosed cardiac conditions in pregnancy and contributes substantially to the elevated rate of maternal mortality [9]. Our study found a point prevalence of 5.0 cases per 1,000 pregnant women. This prevalence is lower than estimates of RHD that were based on a similar case-finding technique in children [5,12], but is comparable to more recent estimates in general adult populations for the world’s RHD endemic regions [19]. A search of the literature found only one other prevalence estimate specific to pregnancy for the burden of RHD disease in sub-Saharan Africa: Otto and colleagues reported a prevalence of 23.0/1,000 women using echocardiography in a case-finding study that included any grade of mitral or aortic regurgitation with valvular thickening [13]. Kenya has a lower RHD prevalence than Eritrea, which could explain some of the discrepancy between these estimates [19]. Additionally, our RHD case definition was based on the World Heart Federation criteria, which require the presence of two associated morphological features in the aortic or mitral valve when assessing regurgitant RHD lesions [11]. Our study population was pregnant women from low-risk antenatal clinics. We did not include women from the high-risk clinic to avoid a selection bias if these women were referred to MTRH specifically because of a diagnosed cardiac lesion. However, this may have contributed to a lower prevalence estimate by excluding women at higher risk of RHD who attended the high-risk clinic for unrelated indications.

The prevalence of non-RHD cardiac lesions was 16.7 per 1,000 pregnant women in this study. These were predominantly valvular lesions that did not meet criteria for RHD; however, other clinically-relevant lesions were also diagnosed. Bicuspid aortic valve (BAV) is a common congenital cardiac malformation. BAV has a wide spectrum of presentations and is often asymptomatic in pregnancy, although this condition carries higher lifetime risks of aortic stenosis, incompetence, dissection, and infective endocarditis [20]. Similarly, patent ductus arteriosus and unroofed coronary sinus lesions may remain asymptomatic, but are relevant given their potential for adverse clinical sequelae.

Although the point prevalence of RHD was lower than expected based on review of the available literature, the overall rate of clinically-relevant cardiac lesions was substantial. Symptoms were infrequently present and not associated with echocardiography findings. In our study sample, if we had performed selective echocardiography screening based on the presence of symptoms or a suspicious past medical history over two-thirds of cases (69%) would have been missed. Antenatal care providers working in RHD-endemic regions may use a combination of factors in establishing a pretest probability for their patients to determine the utility of diagnostic testing. This study suggests that an absence of characteristics associated with RHD is not necessarily reassuring. This study did not include findings from clinical examination or auscultation in the screening protocol as we were primarily interested in cardiac lesion prevalence as estimated by echocardiography. However, clinical exam features may assist clinicians in determining which patients are at higher risk of RHD or other lesions and in selecting additional investigations in the diagnostic workup.

Women with RHD face significant challenges during pregnancy. Adverse maternal outcomes occur in over one-third of cases and fetal or neonatal complications—including perinatal death—reach 30% in registries reporting mainly on middle- and high-income settings [2,18,21]. Faced with these elevated risks, emphasis must be placed on prevention, early detection, and timely management. A concerning finding from our study was the lack of patient awareness regarding cardiac history. Of the 27 (4.5%) women who described a suspected cardiac condition, none were familiar with the name or type of condition and only 23% of women found to have significant echocardiography findings identified any history suggestive of cardiac conditions. This phenomenon has been reported previously and likely reflects the interaction of poverty, low education, and reduced access to resources and medical care [6]. Though not a primary focus of the study, we found that all cardiac lesions were identified in women with secondary school education or lower. It is unclear whether this represents a truly higher rate in women with limited educational opportunities or better access to care and subsequent RHD detection among women with post-secondary educational attainment. It is also possible that symptomatic women from higher socioeconomic backgrounds were more likely to seek tertiary referral for known or suspected cardiac conditions including the high–risk obstetrics clinic at MTRH. Either way, this finding reiterates the concerning social gradient in RHD disease. Conversely, HIV infection and private medical insurance were not associated with RHD or other cardiac lesions. Since 2013, maternal healthcare has been funded by the Kenyan government under the Free Maternity policy [22]. It is possible that better access to medical care is now available among groups historically marginalized based on these and other social determinants of health.

This study demonstrates that screening echocardiography performed by a skilled cardiac sonographer in a low-risk antenatal population is acceptable to women. The average duration for each scan was short, supporting the feasibility of an echocardiography-based screening program. However, our study had access to a skilled cardiac sonographer, research nurse, and separate screening area to perform scans. These resources may not be widely available, particularly in community settings. Likely, a combination of aggressive treatment of childhood streptococcal pharyngitis, pre-pregnancy education around the antecedents and symptoms of valvular heart disease, and a low clinical pretest probability threshold for screening echocardiography are needed to reduce rates of RHD in the pregnant population [3,21,23]. The cost-effectiveness of echocardiography screening for pregnant women was outside the scope of this study and represents an important area for future research.

RHD continues to contribute substantially to maternal and perinatal morbidity. Endemic disease is much more likely in low-income settings and a disproportionate burden is attributed to populations living in poverty and with poor access to care [3,21,24]. This study adds to the available knowledge about RHD prevalence in a population of women with low-risk pregnancies in Kenya. Future efforts should be directed at cost-effective screening strategies in RHD endemic settings, particularly during pregnancy.

### 4.2 Strengths and limitations

We report a point prevalence of RHD using a predetermined standardized set of echocardiography criteria applied to a prospective sample of women recruited from a general antenatal clinic. Screening did not rely on reported symptoms or clinical assessment to identify pretest risk of RHD, thereby, avoiding verification bias. Assessments were performed by a single cardiac sonographer with experience working at MTRH. This minimized bias attributable to interrater differences in the evaluation or interpretation of echocardiography results. The study benefitted from excellent prospective recruitment and thorough capture of demographic, pregnancy, and past medical history information.

We nonetheless experienced some limitations, primarily related to the identification of RHD and other cardiac lesions from only a low-risk antenatal population. Pregnant women already being treated for a previously diagnosed cardiac condition are referred directly to the high-risk antenatal clinic at MTRH. To avoid selection bias, women from this clinic were not eligible for this study. However, women with other non-cardiac conditions are also referred directly to the high-risk clinic and as such may have been excluded despite being part of the population-at-risk. If any, we expect that this selection effect would result in an underestimation of the baseline RHD prevalence. Our RHD case definition was based on international echocardiography screening criteria. Limitations of this approach have been pointed out previously, including the reliance on a skilled cardiac sonographer, and the exclusion of clinical signs and symptoms used in other diagnostic criteria for RHD [11,19].

## 5. Conclusion

This study found an elevated prevalence of clinically-relevant cardiac lesions in pregnant women presenting for routine antenatal care to a large maternal referral hospital in Western Kenya. It demonstrates the acceptability of screening echocardiography among pregnant women. Future work should evaluate whether routine screening echocardiography could be effectively implemented in low-income settings and whether such screening reduces the rate of RHD-attributable maternal mortality.

## Additional Files

The additional files for this article can be found as follows:

10.5334/gh.826.s1 Appendix 1. Echocardiographic Screening Protocol.

10.5334/gh.826.s2 Appendix 2. Echocardiography worksheet.
