# Supplementary material for: Prevalence of Rheumatic Heart Disease and Other Cardiac Conditions in Low-Risk Pregnancies in Kenya: A Prospective Echocardiography Screening Study
Source: Glob Heart. 2021 Feb 9;16(1):10. doi: 10.5334/gh.826 (PMC7879998; doi:10.5334/gh.826)
Supplement: Appendix 1. — Echocardiographic Screening Protocol. [file gh-16-1-826-s1.pdf]

Appendix 1. Echocardiographic Screening Protocol

Pathologies identified requiring further evaluation or treatment will be referred to the MTRH CDU.

1. **Parasternal long axis**
    a. 2D showing Left Atrium (LA), Left Ventricle (LV), Mitral Valve (MV), Aortic Valve (AoV), Right Ventricle (RV) and pericardium
    b. Color Doppler (CD) of MV
    c. CD of AoV
2. **Parasternal short axis**
    a. 2D at Papillary muscle(s) level
    b. 2D at MV level
    c. 2D at Great vessels level
    d. CD of Right ventricular outflow tract/Pulmonary valve(PV)
    e. CD of Interatrial septum
    f. 2 CD sweeps of Ventricular septum
3. **Apical 4 chamber view**
    a. 2D showing LV, LA, RV, RA
    b. CD of TV
    c. CD of MV
4. **Apical 5 chamber view**
    a. CD of AoV
5. **Subcostal view**
    a. Inferior Vena Cava size during respiratory cycle
6. **Suprasternal Notch**
    a. 2D long axis of Aortic arch
    b. CD of descending part of Aortic arch

Additional images may be acquired to further assess suspected pathology.

RHD case-definition[1]

Echocardiographic criteria for definite RHD (at least one of A, B, C, or D):
A) Pathological MR and at least two morphological features of RHD of the MV
B) MS mean gradient ≥4 mmHg
C) Pathological AR and at least two morphological features of RHD of the AV
D) Pathological AR and at least two morphological features of RHD of the MV

Mitral valve morphological features of RHD:
- Anterior mitral valve leaflet thickening ≥3 mm
- Chordal thickening
- Restricted leaflet motion
- Excessive leaflet tip motion during systole

Aortic valve morphological features of RHD:
- Irregular or focal thickening
- Coaptation defect
- Restricted leaflet motion
- Prolapse

[1]Adapted from Reményi et al. World Heart Federation criteria for echocardiographic diagnosis of rheumatic heart disease—an evidence-based guideline. Nat. Rev. Cardiol. 2012; 9, 297–309
